# Informed push distribution of contraceptives in Senegal reduces stockouts and improves quality of family planning services

**DOI:** 10.9745/GHSP-D-13-00171

**Published:** 2014-05-13

**Authors:** Bocar Mamadou Daff, Cheikh Seck, Hassan Belkhayat, Perri Sutton

**Affiliations:** a Ministry of Health and Social Action [Senegal], Dakar, Senegal; b IntraHealth International, Senegal Urban Reproductive Health Initiative, Dakar, Senegal; c McKinsey & Company, Casablanca, Morocco; d Bill and Melinda Gates Foundation, Seattle, WA, USA

## Abstract

Contraceptive use in Senegal is among the lowest in the world and has barely increased over the past 5 years, from 10% of married women in 2005 to 12% in 2011. Contraceptive stockouts in public facilities, where 85% of women access family planning services, are common. In 2011, we conducted a supply chain study of 33 public-sector facilities in Pikine and Guediawaye districts of the Dakar region to understand the magnitude and root causes of stockouts. The study included stock audits, surveys with 156 consumers, and interviews with facility staff, managers, and other stakeholders. At the facility level, stockouts of injectables and implants occurred, on average, 43% and 83% of the year, respectively. At least 60% of stockouts occurred despite stock availability at the national level. Data from interviews revealed that the current “pull-based” distribution system was complex and inefficient. In order to reduce stockout rates to the commercial-sector standard of 2% or less, the Government of Senegal and the Senegal Urban Reproductive Health Initiative developed the informed push distribution model (IPM) and pilot-tested it in Pikine district between February 2012 and July 2012. IPM brings the source of supply (a delivery truck loaded with supplies) closer to the source of demand (clients in health facilities) and streamlines the steps in between. With a professional logistician managing stock and deliveries, the health facilities no longer need to place and pick up orders. Stockouts of contraceptive pills, injectables, implants, and intrauterine devices (IUDs) were completely eliminated at the 14 public health facilities in Pikine over the 6-month pilot phase. The government expanded IPM to all 140 public facilities in the Dakar region, and 6 months later stockout rates throughout the region dropped to less than 2%. National coverage of the IPM is expected by July 2015.

## Abstract (precis)

Dedicated logisticians restocked contraceptives monthly at facilities to maintain defined minimum stock levels, freeing up clinic staff. High stockout rates were virtually eliminated. Also, quality and timely data on contraceptives distributed allowed for better program management.

## CONTEXT

Family planning use in Senegal is among the lowest in the world and has barely increased over the past 5 years, from 10% among married women in 2005 to 12% in 2011.^1^ This low modern contraceptive prevalence rate (MCPR) is an important limiting factor for the country to achieve Millennium Development Goal 5 (improve maternal health), given a current maternal mortality ratio of 392 per 100,000 live births.^1^

Unmet need for family planning is high, estimated at 29%.^1^ In other words, nearly 1 currently married woman of every 3 wants to delay her next birth or stop childbearing entirely but is not using contraception. In the Dakar region, where almost one-quarter of the country's population lives, unmet need is even higher, at 32%, despite a higher MCPR of 21%. Recognizing the critical importance of addressing unmet need for family planning, the Government of Senegal set an ambitious goal to increase MCPR among married women from 12% to 27% between 2011 and 2015, which would result in approximately 350,000 additional family planning users.

In support of this goal, the Bill and Melinda Gates Foundation is providing funding to IntraHealth International to implement the Urban Reproductive Health Initiative (URHI) in Senegal, a collaboration with government and nongovernmental partners to demonstrate that improving the quality of integrated maternal and reproductive health services and expanding the role of the private sector in providing family planning can significantly increase contraceptive use. URHI is testing innovative approaches to improve quality of and expand access to family planning information, supplies, and services, with a goal of achieving a 20 percentage point increase in modern contraceptive uptake in urban areas of Senegal.

Early on in the project, it became apparent that contraceptive stockouts in public facilities (where 85% of women access family planning services^1^) severely limited URHI's ability to test service delivery and demand creation interventions. More critically, anecdotal evidence indicated that women who sought family planning services at health facilities were frequently denied their preferred contraceptive method due to stockouts.

This article reviews results of a supply chain study to understand the magnitude and root causes of stockouts. It also provides an overview of a new distribution system pilot-tested in 1 district of Dakar and the effects of that system on stockout rates and overall management practices.

## METHODS

We conducted a study between July 2011 and December 2011 of the in-country supply chain serving public-sector facilities in the adjacent districts of Pikine and Guediawaye in the Dakar region. At the time of the study, Pikine had a total population of approximately 375,000, served in the public sector by 3 obstetricians/gynecologists and 39 midwives across 1 health center and 13 health posts. Guediawaye had a total population of approximately 333,000, served in the public sector by 3 obstetricians/gynecologists and 35 midwives across 1 health center and 18 health posts.

The study included contraceptive stock audits over a 6-month period and a review of the previous 12 months of stock data at these 33 public-sector health facilities as well as at the district, regional, and national warehouses. In addition, we surveyed 156 consumers and interviewed facility staff, health system managers, government leaders, and donors.

A contraceptive stockout was defined as zero units available for sale at the facility on a day when the facility was open. To derive an annual percentage rate of stockouts, the number of days that a product was stocked out at a facility over a 1-year period was divided by 261 days of operation for that facility.

## RESULTS

According to facility audits, stockouts of injectable contraceptives occurred, on average, 43% of the year. For this method, which requires 4 injections per year for effective contraceptive protection, a 43% stockout rate poses a significant barrier to continuous and sustained use. For contraceptive implants, the stockout rate was almost twice as high, at 83% of the year.

Stockouts of injectables occurred 43% of the year, and of implants, 83% of the year.

Through interviews with women currently using contraceptives, 84% reported that they had experienced a stockout of their preferred method in the past year. Among women experiencing a stockout, 55% switched methods (often to a less effective method), and 45% either discontinued use or went to the private sector and paid 3 to 9 times the price they would have paid at the public-sector facility.

Based on the warehouse and facility audits, we estimated that at least 60% of stockouts occurred despite stock availability at the national level, indicating that the in-country distribution system was not designed well or functioning effectively. Interviews and field observations revealed the following key issues:

- The public-sector distribution system is a highly complex “pull-based” system, involving an excessive number of steps and relying on about 900 midwives at service delivery points (SDPs) to accurately forecast, track, and order contraceptives. There are several problems with relying on midwives to manage stocks: the midwives lacked training on and ownership of the process, and more importantly, they lacked time because they were busy providing reproductive, maternal, and child health services.
- The pull-based system requires health facilities to replenish supplies using their own cash on hand, that is, working capital. In practice, most facilities have limited working capital, and so they are compelled to prioritize commodities that generate higher margins for the facility. Contraceptives generate relatively low margins, and so they are not a high priority, resulting in either limited or no replenishment of the facility's contraceptive stock.
- Facilities are responsible for picking up supplies from warehouses at their own expense, and frequently health care providers must take time away from providing services to perform this task.
- Many facilities maintain poor inventory records, thus providing little visibility into contraceptive method preference and consumption. The lack of accurate and timely data limits the country's ability to monitor and manage the performance of the family planning program.

At least 60% of stockouts occurred despite stock availability at the national level.

The standard “pull-based” system required health facilities to replenish supplies using limited cash on hand.

## KEY MEASURES FOR IMPROVEMENT

With clear evidence and data on the magnitude of stockouts and the associated impact on individual and population health indicators, the government became highly motivated to reduce stockout rates to the commercial-sector standard of 2% or less. In addition, reliable data on product consumption across methods and facilities were critical to monitor the performance of the supply chain and to understand service delivery patterns.

## STRATEGY FOR CHANGE

To achieve our goal of an aggressive reduction in stockouts, we analyzed commercial-sector solutions and applications of those solutions in public health systems. The “Delivery Team Topping Up” system in Zimbabwe,^2^ a successful example of a widely practiced vendor-managed inventory model, provided a foundation on which to build a model adapted to Senegal's environment and needs.

**FIGURE 1.**
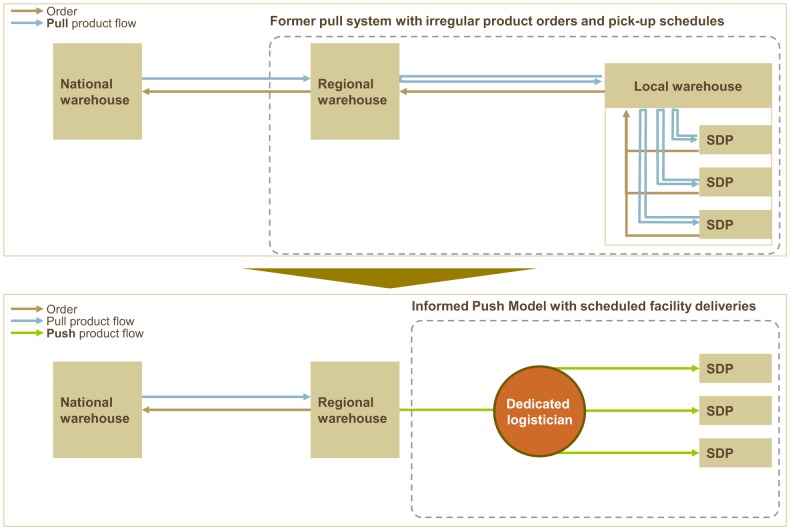
Informed Push Model Streamlines Deliveries and Eliminates Orders Between Service Delivery Points (SDPs) and the Regional Warehouse

URHI and the Government of Senegal developed the “informed push distribution model” (IPM) and tested it in Pikine district over a 6-month pilot phase from February 2012 to July 2012. IPM brings the source of supply (a delivery truck loaded with supplies) closer to the source of demand (clients in health facilities) and streamlines the steps in between. With a professional logistician managing stock and deliveries, the health facilities no longer need to place orders and spend time picking up products (Figure 1).[Fig f06]

The informed push model brings the source of supply closer to the source of demand.

Key features of the IPM include:

- Initial stock of contraceptives is provided at no cost to each facility.
- Dedicated logisticians restock facilities on a monthly basis to maintain a minimum level of stock that is defined by the logistician—around 2 months of estimated supply needs.
- Facilities pay only for the quantity of products that were sold and keep the margin.
- Logisticians collect data on product consumption at the time of delivery and report that data to the district medical chief within 72 hours.
- Logisticians are paid according to fixed-fee contracts that clearly define requirements and penalties based on stockout rates and data availability. During the pilot study, logistician contracts were funded and managed by the URHI program.
- Contraceptive sales revenue covers the cost of the logistician contracts.

**Figure f06:**
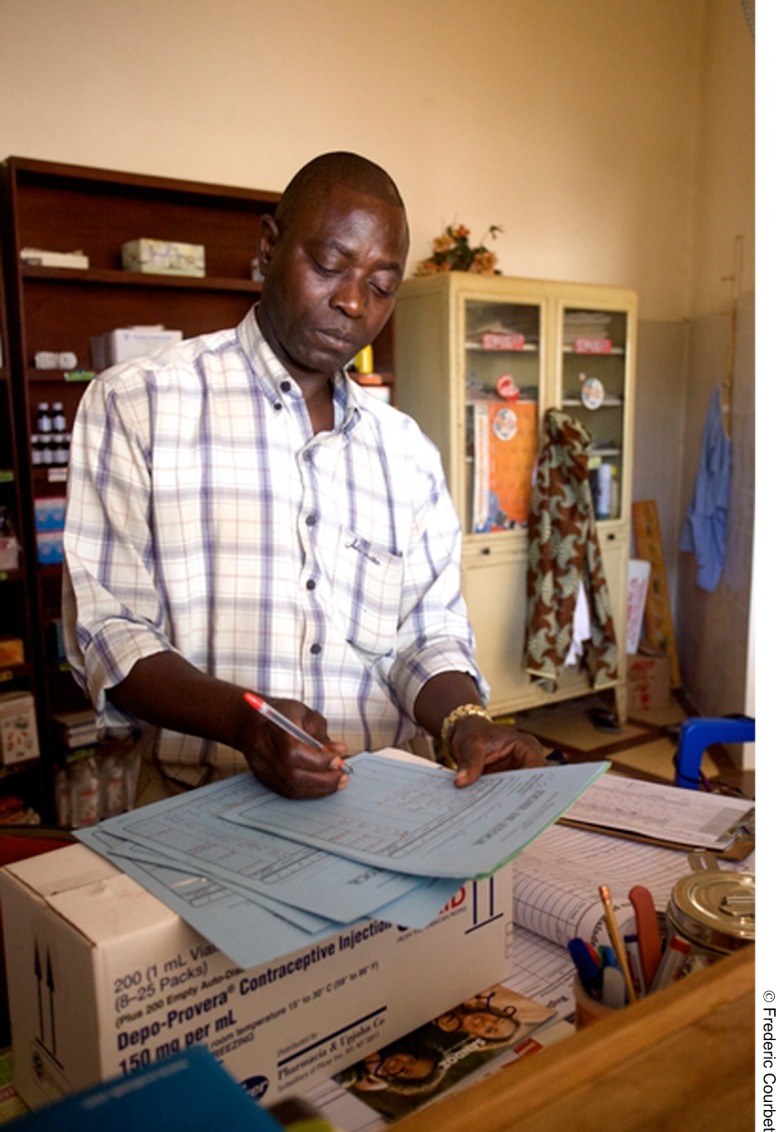
A private logistician delivers contraceptives and collects data at a health post in Pikine, Senegal.

## EFFECTS OF CHANGE

**FIGURE 2.**
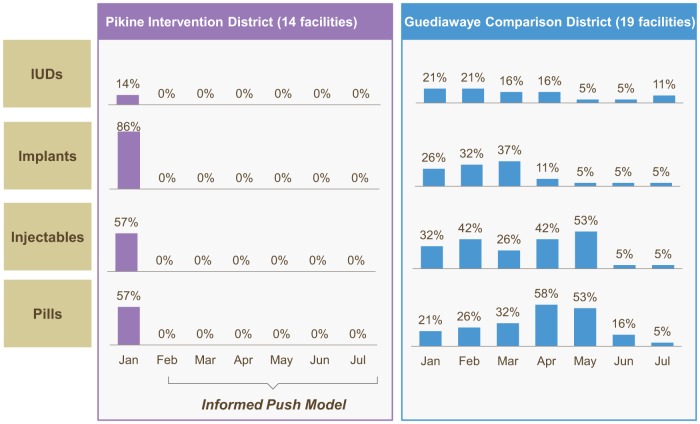
Percentage of Facilities Experiencing a Stockout in 2 Comparison Districts, Dakar, Senegal, January–July 2012 Abbreviation: IUDs, intrauterine devices.

The effects of IPM implementation in Pikine were immediate and sustained. Stockouts of contraceptive pills, injectables, implants, and intrauterine devices (IUDs) were completely eliminated at all 14 public health facilities in the district over the course of the 6-month pilot phase. In the month before IPM was implemented, injectables and pills were stocked out in 57% of facilities, implants were stocked out in 86% of facilities, and IUDs in 14% (Figure 2). During the same pilot period, in the neighboring district of Guediawaye where IPM was not implemented, stockouts of these products persisted, on average, at 23% of facilities.

During the informed push pilot phase, contraceptive stockouts were completely eliminated.

Based on this dramatic impact on stockouts, the government decided to expand IPM to all 140 public facilities in the Dakar region; 6 months later, stockout rates throughout the region dropped to less than 2% according to facility and delivery records.

Importantly, district and regional health managers now have access to timely and accurate monthly contraceptive consumption data from each participating SDP, as required by the logistician contracts, which enables the managers to quickly identify and address performance issues. For example, the Pikine district health management team identified a clinic that consistently consumed fewer implants than neighboring clinics. During a follow-up visit, the team learned that the midwife responsible for providing implants was uncomfortable with the procedure and unmotivated to learn it since she was nearing retirement. The management team responded by prioritizing this site to receive a newly graduated midwife; once placed, implant consumption increased to a level similar to that recorded in neighboring clinics.

Better data from the informed push model have helped managers address performance issues.

In another example of performance management enabled by the IPM data, the district noticed a significant decline in implant consumption across facilities in a single month. The management team quickly learned that there was a shortage of the local anesthetic used for the implant procedure, which triggered an emergency order at the national level.

**FIGURE 3.**
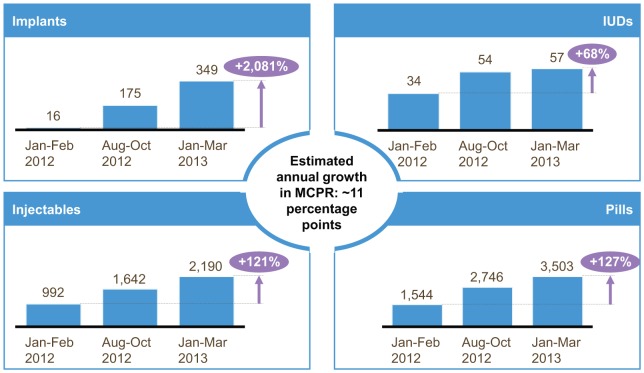
Average Monthly Consumption of Contraceptives in Pikine District, Dakar, Senegal, Before and 1 Year After IPM Implementation Abbreviations: IPM, informed push model; MCPR, modern contraceptive prevalence rate.

In Pikine, after 1 full year of implementing the informed push model (the 6-month pilot plus the following 6 months during expansion to the entire Dakar region) and maintaining stock of the full range of contraceptive methods, coupled with demand creation interventions and service delivery improvement measures, the average monthly consumption of pills increased by 127%, injectables by 121%, implants by 2,081%, and IUDs by 68%. These increases translate to an estimated growth in MCPR of approximately 11 percentage points in 1 year in Pikine, an unprecedented rate of growth in Senegal (Figure 3).

**FIGURE 4.**
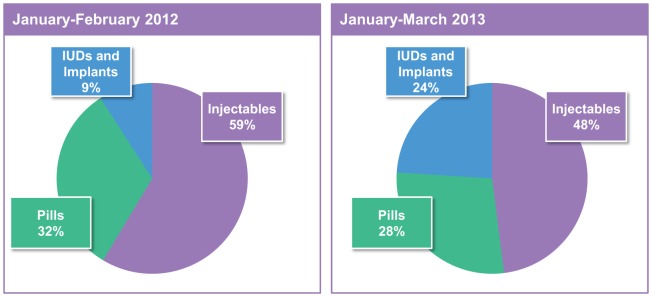
Method Mix^a^ Before and 1 Year After IPM Implementation, Pikine District, Dakar, Senegal Abbreviations: IPM, informed push model; IUDs, intrauterine devices. ^a^ Excluding condoms.

When all methods are available at a facility, health care providers are more comfortable counseling women on the full range of options, and the woman has the opportunity to select the method she prefers without the influence of stockouts. In fact, since stockouts have been eliminated in Pikine, the method mix has evolved with a greater proportion of women selecting long-acting methods (Figure 4).

In addition, other positive changes resulted from IPM implementation. First, health care workers have more time to provide care to patients because they do not have to manage stocks and logistics. Second, the regional and district health managers who experienced the impact of IPM for contraceptive products are advocating to use this distribution system for additional products. One example of where this has worked is in the region of Saint Louis, where 2 mobile warehouses were in place to deliver vaccines. The IPM was adapted to use the mobile warehouses for contraceptive distribution and data collection, and subsequently the regional managers insisted that HIV, malaria, tuberculosis, and essential medicines were also delivered via the mobile warehouses. This adapted version of IPM requires additional study to determine its cost-effectiveness relative to the contraceptive-only model implemented in Pikine.

The informed push model has freed up health care workers to spend more time providing care to patients.

## NEXT STEPS

**FIGURE 5.**
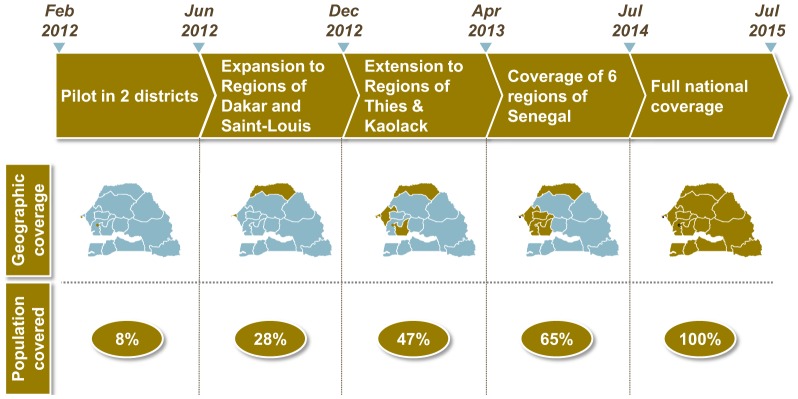
National IPM Scale-Up Plan, Senegal Abbreviation: IPM, informed push model.

In 2012, the Ministry of Health developed and launched a national family planning plan. Given the positive IPM results from Pikine, one of the key pillars of the plan is national expansion of the IPM. By July 2014, the plan calls for scaling up IPM throughout the 6 regions that comprise more than 65% of the population, and to national coverage by July 2015 (Figure 5).

Senegal plans to scale up the informed push model nationally by 2015.

To support rapid national expansion, the Bill and Melinda Gates Foundation and Merck for Mothers are providing funding to IntraHealth International to support the Government of Senegal to establish standard operating procedures; issue and manage contracts with private logisticians; provide training to those engaged in the system; and support data use and performance management. The IPM is currently designed to maintain preexisting financial flows to the extent possible while introducing payment of contracts with private logisticians. As the model expands into regions that are less densely populated, and with more difficult road conditions, modifications to the model are expected to enable delivery optimization. According to an agreed-upon schedule through mid-2016, these functions will be funded and overseen by the government in a phased manner.

To ensure financial sustainability of the IPM, the government is evaluating different scenarios, and IPM design will remain flexible to respond to the most cost-effective and politically viable option. At present, the government is committed to advancing implementation of the IPM for family planning products, and there is interest in conducting an in-depth product segmentation analysis to guide the potential inclusion of other health products. Preliminary analysis suggests that the annual sales revenue from contraceptives when volumes reach the level required to support a national MCPR of 25% to 30% will be about US$1,050,000. At a national scale in the contraceptive-only scenario, the operating cost of the IPM was estimated at about $500,000, approximately 11% of national annual spending on contraceptives.^3^ While further study to refine the cost estimates is needed, a number of strategies to maintain funding for the IPM at national scale are under consideration, for example, using revenue from contraceptive and other product sales, including a government budget line item for product distribution, and collaborating with other donors and multilateral programs supporting product distribution.

Today, the public health system in Senegal is served by 7 unique supply chains, one for each of the 7 different product groups. Each vertical supply chain is funded through a unique agreement between a donor, the Ministry of Health, and the National Pharmacy, resulting in a wide range of operating procedures, personnel trainings, supervisory structures, data collection tools, and reporting requirements. The net effect is a distribution system that is uncoordinated and inefficient, resulting in stockouts across all product groups. With the political will and donor flexibility to better coordinate and manage this multiplicity as a segmented system, cost savings across Ministry of Health programs could be significant. Further analysis of costs associated with the current public-sector distribution channels across product categories is recommended, along with a comparative analysis of the cost per unit of product delivered through alternative distribution models.

## CONCLUSION

This pilot study of the informed push distribution model demonstrated feasibility as an appropriate and effective solution to contraceptive stockouts in Senegal, generating timely and accurate data on contraceptive consumption by facility. Access to and use of this data is transforming public health management practices in general, fostering a culture of data-driven performance improvement throughout the system. Achievement of Senegal's goals for family planning depend on the successful implementation of all priority interventions in the national plan, including demand generation, improved provider capacity, and an expanded network of SDPs; the IPM ensures availability of the products and data that underlie such success.
